# Présentation clinique atypique d’un kyste hydatique thymique primitif: à propos d’une observation Marocaine

**Published:** 2012-03-12

**Authors:** Taoufiq Harmouch, Amal Benlemlih, Nawal Hammas, Mouhcine Bendahou, Abdelatif Oudidi, Mohammed El Alami, Afaf Amarti

**Affiliations:** 1 Laboratoire d’anatomie et de cytologie pathologiques, CHU Hassan II, Fès, Maroc; 2 Service d’ORL, CHU Hassan II, Fès, Maroc

**Keywords:** Hydatidose, Thymus, intrathoracique, cervicale, Maroc

## Abstract

Le kyste hydatique est une affection encore endémique au Maroc. Il peut toucher tous les organes notamment le foie et le poumon. La localisation médiastinale est rare et souvent primitive. Elle représente moins de 1% des localisations intrathoraciques et l’atteinte thymique est exceptionnelle. Nous rapportons un cas de kyste hydatique thymique primitif chez une fille de 9 ans hospitalisée pour une masse cervicale médiane antérieure. Nous étudierons les aspects épidémiologiques, cliniques et les démarches diagnostiques pour cette localisation exceptionnelle du kyste hydatique. Les aspects cliniques, radiologiques et épidémiologiques de l’hydatidose restent des éléments de présomption. L’examen histologique a toute sa valeur devant les localisations inhabituelles.

## Introduction

Le kyste hydatique (KH) est une parasitose largement répandue dans les pays méditerranéens. Elle est due au développement chez l’homme de la larve d’*Echinococcus granulosus* qui est un petit ténia vivant dans l′intestin des chiens. L’homme s’insère accidentellement dans le cycle du parasite dont l′hôte définitif est le chien et l′hôte intermédiaire est le mouton.

Ceci explique la forte incidence de cette pathologie dans les pays d′élevage traditionnel. Après une ingestion du parasite par voie orale, il va suivre le courant sanguin et lymphatique entrainant une atteinte de n’importe quel organe. Dans la littérature, 11 cas de localisation thymique ont été recensés mais aucun ne s’est présenté comme une masse cervicale [1–9].

Les auteurs rapportent un cas de kyste hydatique thymique primitif à localisation cervicale chez une fille de 9 ans diagnostiqué après l’examen histologique de la pièce opératoire.

## Patient et observation

Il s’agit d’une fille de 9 ans issue du milieu rural avec une notion de contact avec les chiens et qui a présenté un an avant sa consultation une masse cervicale indolore augmentant progressivement de volume. L’examen clinique avait révélé une masse cervicale sus-sternale médiane de 5cm de diamètre, ferme, mobile et indolore à la palpation. La peau en regard de la masse est d’aspect normal sans signe de compression. Le tout évoluant dans un contexte de conservation de l’état général.

La tomodensitométrie retrouve une masse arrondie, hypodense et cloisonnée para trachéale antérieure. La patiente a bénéficié d’une cervicotomie exploratrice avec exérèse chirurgicale complète de la masse.

**Figure 1:**
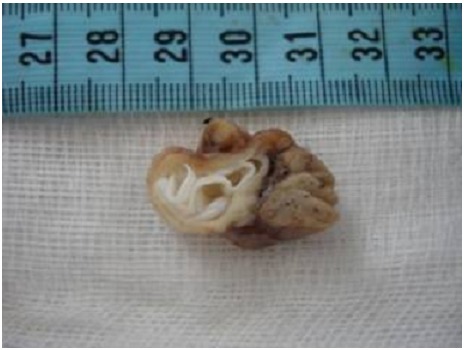
Masse kystique Thymique à paroi blanchâtre légèrement décollée (Membrane Hydatique)

Sur le plan macroscopique on note la présence d’une masse de 4x1.5 cm comportant un nodule de 2x1.3x1 cm. A la coupe, présence de membrane fine blanchâtre (Figure 1).

**Figure 2:**
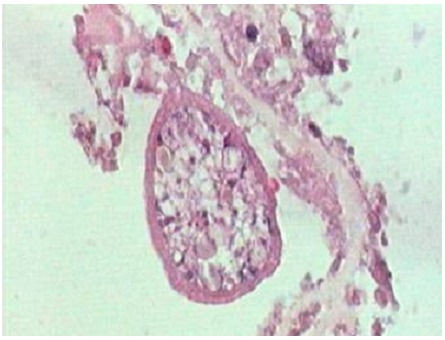
Scolex d’Echinococcus granulosus. HES, Gx400

Sur le plan histologique, il s’agit d’un parenchyme thymique comportant une formation kystique bien limitée, bordée par une paroi anhiste, rubanée et éosinophile. On y retrouve des scolex d’*Echinococcus granulosus* (Figure 2).

Les suites opératoires étaient simples et la patiente n’a pas présenté de récidive ou de localisation secondaire après un recul de 2ans.

## Discussion

Le kyste hydatique répond au développement chez l’homme, à l’état larvaire ou vésiculaire (hydatide) d’un tænia (tænia *Echinococcus granulosus*) qui vit comme l’adulte dans l’intestin du chien. Le kyste hydatique intéresse le foie dans les deux tiers des cas, et accessoirement, le poumon, la rate, le rein, le cerveau, etc. Cette fréquence n’est pas surprenante, car les œufs du parasite, absorbés avec des aliments souillés, libèrent dans l’estomac, des embryons qui franchi les filtres hépatique et pulmonaire, pénètre dans la circulation systémique. L’atteinte thymique est exceptionnelle pouvant s’expliquer par deux mécanismes. Le parasite se fixe au niveau du thymus par les vaisseaux thymiques qui naissent du tronc commun de la sous - Clavière et parfois de la carotide. Le second mécanisme de cette atteinte est celui d’un cheminement par les voies chylifères et lymphatiques.

La paroi du kyste hydatique comporte deux couches. La plus interne, dite couche germinative ou membrane proligère, est nucléée. Elle donne naissance à des capsules ou vésicules proligères, d’abord pédiculées et attachées à elle, en suite elles se détachent et tombent dans la cavité kystique remplie de liquide eau de roche. Ces vésicules filles sont le lieu de formation de scolex hexacanthes, à crochets en épine de rosier. Elles peuvent se rompre et libérer leur contenu dans le liquide hydatique, en une poussière blanche dite sable hydatique. Elles peuvent en outre donner naissance à leur tour à des vésicules-petites-filles. A l’extérieur de la membrane proligère, la seconde couche pariétale est une cuticule anhiste. Le parenchyme thymique, refoulé, comprimé et fibrosé, constitue l’adventice au-delà de laquelle se remarque en période active, un infiltrat inflammatoire histiocytaire, giganto-cellulaire et polynucléaire éosinophile. Le kyste hydatique vieilli peut se calcifié, s’infecter ou se fistulisé.

Le kyste hydatique thymique se manifeste par des signes de compression trachéale ou cave supérieure [10,11] qui doivent attirer l’attention surtout en présence de notion de contact avec le chien et dans les pays d’endémie. La manifestation clinique sous forme d’une masse cervicale chez notre patiente s’est produite, probablement, par extension. Le diagnostic est confirmé par la sérologie qui n’est pas constamment positive [12].

L’imagerie permet de poser le diagnostic positif, de rechercher d’autres localisations ainsi que le suivi du malade. La radiographie standard va mettre en évidence une opacité parfois des calcifications très utile pour porter le diagnostic.

L’échographie constitue l’examen clé en précisant la nature liquidienne de la masse avec un aspect multivésiculaire ou univésiculaire aussi, couplée au doppler elle permet de préciser les rapports de la masse avec les structures vasculaires. Elle objective des aspects comparables à d’autres localisations, notamment hépatiques répondant à la classification de Gharbi dont les types I et IV posent souvent les problèmes de diagnostic différentiel [13].

Dans tous les cas la tomodensitométrie est nécessaire permettant: d’affirmer ou de confirmer le diagnostic, en montrant un non rehaussement de la masse après injection du produit de contraste, une meilleure précision topographique et une étude des rapports du kyste hydatique avec les structures avoisinantes. L’IRM permet de mieux préciser la topographie des kystes et les rapports avec les organes de voisinage grâce aux séquences pondérées T2 permettant une visualisation des vésicules filles ou une membrane flottante.

Le traitement de choix reste la chirurgie avec un risque de rupture du kyste et dissémination du liquide pouvant provoquer un choc anaphylactique avec un risque de récurrence. Pour cela, il faut mettre des champs imbibé de sérum salé hypertonique autour du kyste à réséquer. Le traitement médical peut être utilisant comme traitement adjuvant à la chirurgie, dans le cas d’une hydatidose multiple ou en cas de malade inopérable.

Notre patiente a bénéficié d’une chirurgie seule et n’a pas présenté de récidive ou de localisation secondaire après un recul de 2 ans.La prophylaxie nécessite de couper le cycle, en traitant les chien et en brulant les abas infestés.

## Conclusion

L’hydatidose est une anthropozoonose qui sévit à l’état endémique au Maroc. La symptomatologie clinique est polymorphe et les aspects radiologiques et épidémiologiques restent des éléments de présomption. Devant les localisations inhabituelles, c’est l’examen histologique des pièces opératoires qui permet de poser le diagnostic.
